# Impact of *Helicobacter pylori* Biofilm Formation on Clarithromycin Susceptibility and Generation of Resistance Mutations

**DOI:** 10.1371/journal.pone.0073301

**Published:** 2013-09-06

**Authors:** Hideo Yonezawa, Takako Osaki, Tomoko Hanawa, Satoshi Kurata, Kuniyasu Ochiai, Shigeru Kamiya

**Affiliations:** 1 Department of Infectious Diseases, Kyorin University School of Medicine, Tokyo, Japan; 2 Department of Microbiology, Nihon University School of Dentistry, Tokyo, Japan; Iowa State University, United States of America

## Abstract

The human gastric pathogen *Helicobacter pylori* forms biofilms *in vitro* and *in vivo*. The purpose of this study was to evaluate the effects of *H. pylori* biofilm formation *in vitro* on clarithromycin (CLR) susceptibility. CLR susceptibility of *H. pylori* intermediate (2-day) and mature (3-day) biofilms on glass coverslips was determined at concentrations from 0.03 to 0.5 µg/ml. *H. pylori* biofilm biomass was increased after treatment with CLR at minimum inhibitory concentration levels by up to 4-fold (2-day biofilm) and 16-fold (3-day biofilm). Minimum bactericidal concentrations of CLR against cells in a biofilm were higher (1.0 µg/ml) than that for planktonic cells (0.25 µg/ml). It was shown that the expression of efflux pump genes was significantly increased in biofilm cells. In addition, exposure of biofilms to CLR resulted in high level resistance generation compared to planktonic cells with increased resistance associated with the presence of a point mutation at either position 2142 or 2143 in the domain V loop of the 23S rRNA gene. These results demonstrate that *H. pylori* biofilm formation decreases the susceptibility to CLR and that *H. pylori* CLR resistance mutations are more frequently generated in biofilms than in planktonic cells.

## Introduction

Biofilms are ubiquitous in natural, industrial and clinical environments, and have been shown to be critical in many chronic infections [1]. Biofilm development is initiated when bacteria convert from a planktonic state to a lifestyle in which they are firmly attached to biotic or abiotic surfaces. Biofilm bacteria express several properties distinct from planktonic cells, one of which is an increased resistance to antimicrobial agents and this process is thought to be a major contributor to the etiology of infectious diseases [2].

*Helicobacter pylori* is a spiral, microaerophilic, non-invasive, Gram-negative bacterium that colonizes the human gastrointestinal tract, primarily the stomach [3]. Recently, some studies have alluded to the ability of *H. pylori* to form biofilms *in vitro* [4], [5], [6]. In addition, *H. pylori* can exist in biofilms formed on human gastric mucosa [7], [8], [9]. Our previous study demonstrated that the strain TK1402, isolated from a patient with duodenal and gastric ulcers in Japan had strong biofilm forming ability *in vitro* [10], [11], [12].

As the first-line therapy for *H. pylori* eradication, the combination of a proton pump inhibitor, either clarithromycin (CLR) or metronidazole, and amoxicillin has been established worldwide [13], [14]. In Japan, a combination of a proton pump inhibitor, amoxicillin and CLR is commonly used in first-line eradication therapy [15]. CLR is a macrolide antibiotic that binds to the 50S subunit of the bacterial ribosome and inhibits the translation of peptides, thus preventing the bacteria from growing. However, CLR-resistance is an increasing problem for the first-line therapy of *H. pylori* infection, since the major cause of eradication failure is thought to be the existence of CLR-resistant *H. pylori* [14]–[18]. CLR resistant *H. pylori* are extremely common and the frequency of CLR-resistant clinical isolates ranges from approximately 10 to 30% [19], [20]. Point mutations in the domain V loop of the 23S rRNA gene (commonly an adenine-to-guanine transition at position 2142 or 2143) have been reported as the basis for resistance [15]–[23].

The aim of this study was to investigate the effects of CLR on *H. pylori* biofilms *in vitro*. Furthermore, we compared the generation of spontaneous resistance to various concentrations of CLR in both biofilm and planktonic cells and the mutations in the 23S rRNA gene of *H. pylori* were determined.

## Materials and Methods

### Bacterial Strain

*H. pylori* strain TK1402, isolated from gastric biopsy specimens of a patient with gastric and duodenal ulcers [24], was used in this study. The strain was maintained at −80°C in Brucella broth (Difco, Detroit, Mich) with 20% (vol/vol) glycerol and was cultured under microaerobic conditions at 37°C on Brucella agar plates containing 7% horse serum.

### Assessment of Susceptibility to CLR

*H. pylori* strain TK1402 was grown on Brucella agar plates containing 7% horse serum; the cells were then suspended in fresh Brucella broth supplemented with 7% fetal calf serum (Brucella-FCS) and cultured for 24 h under microaerobic conditions at 37°C. The pre-culture cells were adjusted to an optical density of 1.0 at 600 nm. In a 12-well microtiter plate, 10 µl of pre-cultured cells were inoculated into 2 ml of duplicate serial two-fold dilutions of CLR in Brucella-FCS from 0.001 µg/ml to 1.0 µg/ml. The cultures were incubated under microaerobic conditions at 37°C for 24 h with shaking (80–100 rpm). After incubation, optical densities of the cultures were examined as the means of three independent experiments.

Assessment of biofilm cell susceptibility to CLR was carried out by a similar method as above using 2-day and 3- day biofilms with slight modifications. Briefly, biofilms of *H. pylori* strain TK1402 were grown as previously described [10]. The coverslips with 2-day or 3-day biofilms were removed from the 12-well plate, washed with phosphate-buffered saline (PBS) and placed into fresh plate wells filled with 2 ml Brucella-FCS and 0.5 µg/ml, 0.25 µg/ml, 0.125 µg/ml, 0.063 µg/ml, 0.031 µg/ml, or 0 µg/ml of CLR. The biofilm cells were then incubated for 24 h under microaerobic conditions at 37°C with shaking. After incubation, the biofilms were assessed with the biofilm assay previously described [10].

### Determination of Cell Viability

To determine the number of viable cells after exposure to CLR, 2-day or 3-day biofilm coverslips were transferred to 2 ml of fresh Brucella-FCS with two-fold dilutions of CLR at concentrations ranging from 0.03 µg/ml to 0.5 µg/ml. Two-day or 3-day planktonic cultures of cells exposed to the same concentrations of CLR were used as controls. After exposure, the biofilm cells were scraped and removed with PBS following mechanical treatment. The colony-forming unit (CFU) values of the cell suspension was then determinbed by plating onto Brucella agar supplemented with 7% FCS (Brucella-FCS agar). The CFUs of the 2-day or 3-day planktonic cell cultures were also evaluated using the same method. Colony numbers were counted after 96 h incubation.

To confirm the cell morphology after treatment with CLR, a scanning electron microscope (SEM) examination was carried out. The CLR exposed 3-day biofilms on the coverslips were fixed with 2% glutaraldehyde for 3 h at room temperature. The samples were observed using a JSM-5600LV electron microscope (JEOL, Tokyo, Japan).

### Extraction of RNA and Real-time Quantitative RT-PCR

To analyze the expression of mRNA in the *H. pylori* biofilms, the 3-day biofilm cells of strain TK1402 were scraped into PBS. Planktonic cells were also cultivated for 3 days at 37°C. After washing of the cells with PBS three times, total RNA extraction was carried out using the RNeasy minikit (QIAGEN GmbH, Hilden, Germany). The RNA samples were then treated with a TURBO DNA-free™ Kit (Applied Biosystems, Foster City, CA) according to the directions of the supplier. Reverse transcription (RT) was carried out with the PrimeScript™ RT reagent Kit (Takara Bio INC. Shiga, Japan) according to the directions of the supplier. Real-time RT-PCR was performed with the cDNA samples with either 16S rRNA-specific primers (Primer Hp16S F; 5′-GAAGATAATGACGGTATCTAAC; R; 5′-ATTTCACACCTGACTAT) [25], or efflux pump-specific primers (HP609 F; 5′-AGCGCAAGAACTCAGTGTCA, R; 5′-GCTTGGAGTTGTTGGGTGTT, HP971 F; 5′-TTACCGGCAAAGGGATACG, R; 5′-AAATTGGATCGCTCGTTGTATG, HP1327 F; 5′-GCCAGGCTTGATGAAGAAAA, R; 5′-TTAGCCTGCTTGCCGTAAAT, or HP1489 F; 5′-TAGGCGCTCAAGTGGCTTAT, R; 5′-TCAGATCGGGCAGATTTTTC) [26], with the SYBR^R^ Premix Ex Taq (Perfect Real Time) Kit (Takara Bio INC.) in an ABI PRISM 7500 Real-time PCR system (Applied Biosystems). The final results were expressed as the levels of the expression of each efflux pump gene relative to that of the 16S rRNA gene.

### Generation of CLR Resistant Mutants

*H. pylori* strain TK1402 biofilm cells were subjected to a passage experiment to generate CLR resistance. The coverslips with 2-day or 3-day biofilms were transferred to 2 ml of fresh Brucella-FCS containing CLR at concentrations of 0.125 µg/ml, 0.25 µg/ml or 0.5 µg/ml. The coverslips with the biofilms were then incubated for 24 h under microaerobic conditions at 37°C with shaking. After incubation, the biofilm cells on the coverslips were mechanically scraped, resuspended into PBS, and the cells were recovered following incubation on Brucella-FCS agar plates for 72 h. All of surface growth after 72 h of incubation was transferred with a swab onto antibiotic free agar plates for isolation and also onto plates containing 1.0 µg/ml of CLR to confirm the generation of CLR resistant cells. If no CLR resistant cells were detected, this process was repeated at least 5 times or until thegeneration of CLR resistant cells was detected.

The same method was used for planktonic cells with some modifications. *H. pylori* was cultured for 48 h (2-day culture planktonic cells) or 72 h (3-day culture planktonic cells). Since our previous study noted that the optical density of scraped biofilms were approximately 0.14 or 0.26 for 2-day and 3-day biofilms, respectively [10], the cultures were adjusted to an optical density of 0.14 for 2-day or 0.26 for 3-day planktonic cells in 2 ml of Brucella-FCS containing 0.125 µg/ml or 0.063 µg/ml of CLR in a 12-well microtiter plate. After incubation, the cells were collected by centrifugation and washed with PBS. The cells were recovered and the generation of CLR resistance was determined as described above.

### DNA Sequencing of the 23S rRNA Domain

The CLR resistant colonies were picked from CLR free Brucella-FCS agar plates and genomic DNA was extracted using MagExtractor™ (TOYOBO CO., LTD. Osaka, Japan) according to the instructions of the supplier. Genomic DNA served as the template for PCR using a specific primer pair to the domain V loop of the 23S rRNA gene (primer Hp23S 1942F; 5′-AGGATGCGTCAGTCGCAAGAT; Hp23S 2308R; 5′-CCTGTGGATAACACAGGCCAGT) [27]. Nucleotide sequences were analyzed directly from purified PCR products. Sequencing reactions were performed in a BioRad DNA Engine Dyad PTC-220 Peltier Thermal Cycler using ABI BigDye ™ Terminator v3.1 Cycle Sequencing Kits with AmpliTaq DNA polymerase (FS enzyme) (Applied Biosystems, Foster City, CA), following the protocols supplied by the manufacturer. Single-pass sequencing was performed on each template using Hp23S 1942F and Hp23S 2308R primers with an ABI 3730xl sequencer (Applied Biosystems).

### Statistical Analysis

Statistical analysis was performed using the Mann-Whitney U test. P values of 0.05 or less were considered to indicate statistical significance.

## Results

### Susceptibility of Strain TK1402 in Broth Culture to CLR

**Figure 1:**
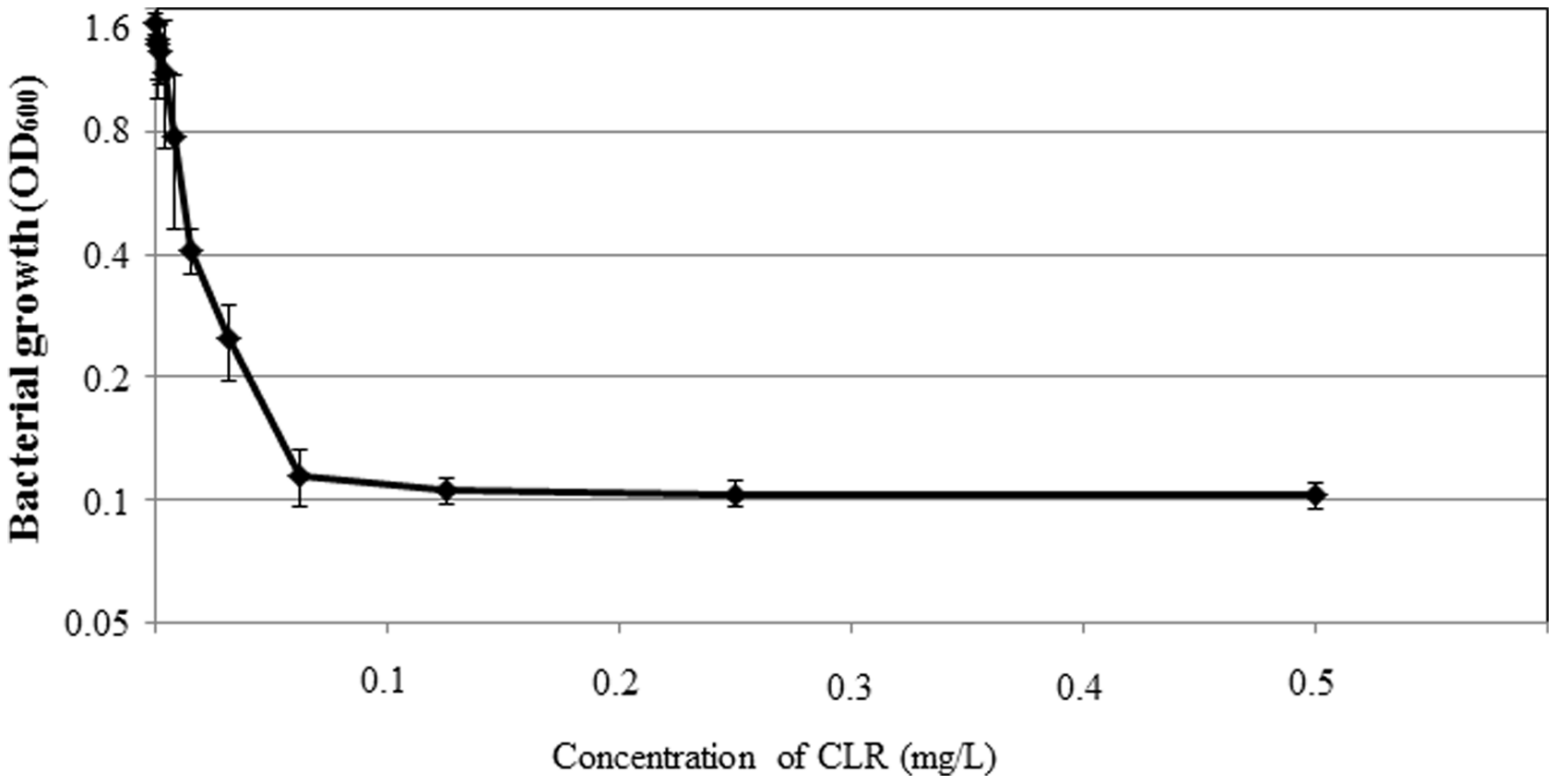
Growth kinetics of *H. pylori* strain TK1402 with CLR. Pre-cultured cells were grown in Brucella-FCS for 24 h with each concentration in a range of 0.5 µg/ml to 0.001 µg/ml or 0 µg/ml of CLR. After incubation for 24 h under microaerobic and shaking condition at 37°C, the optical densities of the cultures were determined. All of the results were expressed as the means ±1 standard deviation from at least three independent experiments.

Since we used broth media for analyzing the CLR susceptibility of biofilm cells in our experimental model, broth microdilution minimum inhibitory concentration (MIC) determinations were carried out using two-fold serial dilutions of the compound with approximately 5×10^5^ cells of initial inoculum in 2 ml of Brucella-FCS (Fig. 1). The cells could not grow in broth media at CLR concentrations of 0.03 µg/ml, whereas the cells grew significantly at 0.016 µg/ml of CLR, indicating that the MIC to CLR under this condition is approximately 0.016 µg/ml. The Clinical and Laboratory Standard Institute defined resistance of *H. pylori* isolates to CLR as MIC of >1 µg/ml, indicating that strain TK1402 was susceptible to CLR.

### Susceptibility of Strain TK1402 Biofilms to CLR

**Figure 2:**
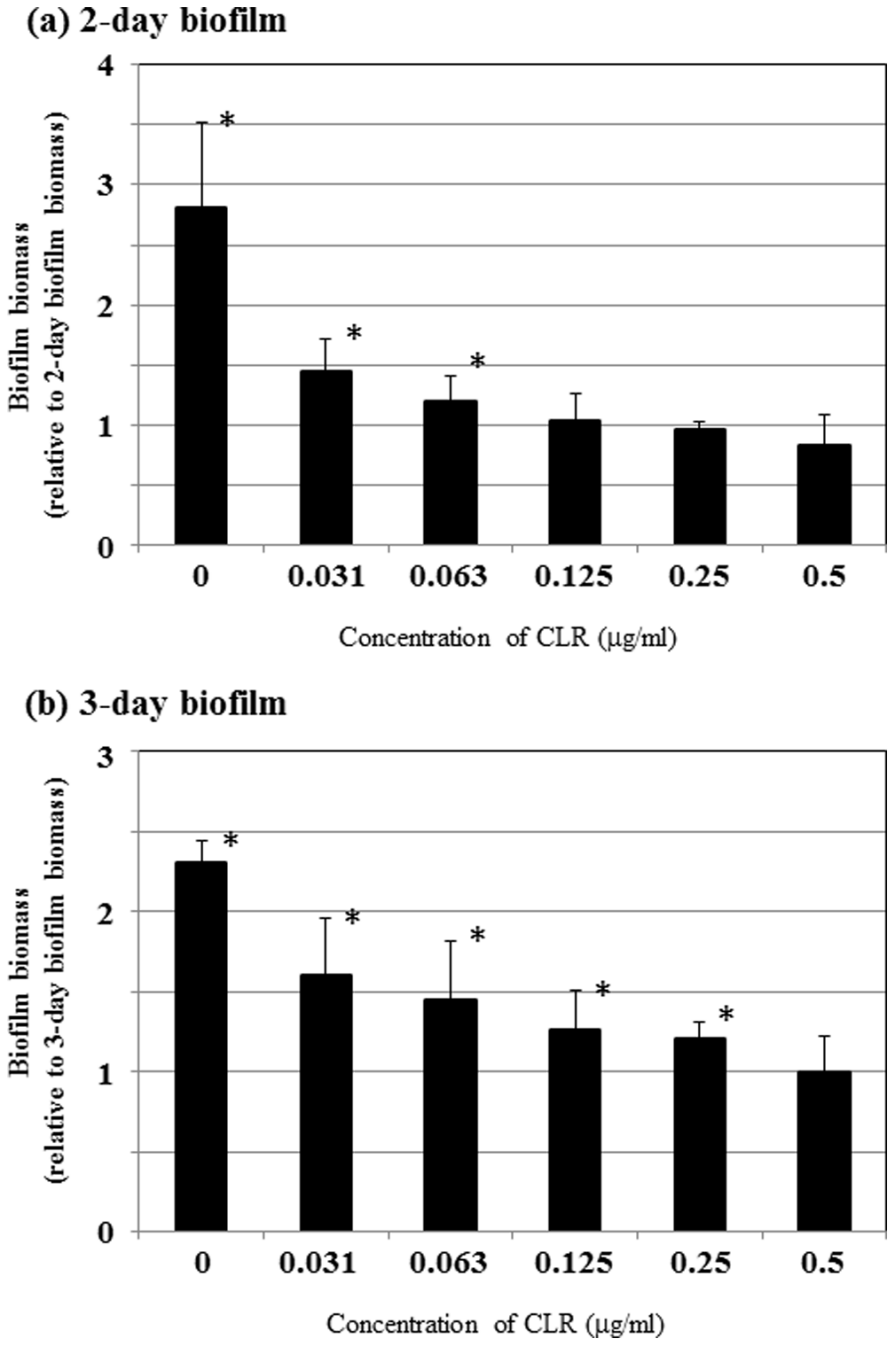
Effect of CLR on strain TK1402 biofilms. The 2-day (a) and 3-day (b) biofilms were transferred into fresh Brucella-FCS with each concentration (0.5 µg/ml, 0.25 µg/ml, 0.125 µg/ml, 0.063 µg/ml, 0.031 µg/ml or 0 µg/ml) of CLR. After incubation for an additional 24 h under microaerobic and shaking conditions at 37°C, the biofilm biomass was measured with crystal violet. The biofilm biomass was calculated relative to starting biofilm biomass (0.53 and 1.51 for 2-day and 3-day biofilm, respectively), which was set at 1.0. All of the results are expressed as the means ±1 standard deviation from at least three independent experiments.*significantly different (*p*<0.05) relative to the level of starting biofilm biomass (starting biofilm biomass versus after biofilm biomass exposure to CLR).

In order to examine the susceptibility of strain TK1402 biofilms to CLR, the 2-day or 3-day biofilms were exposed to CLR at concentrations ranging from 0.031 µg/ml to 0.5 µg/ml, which are concentrations equivalent to 2×MIC to 32×MIC. In the biofilm assay with strain TK1402, the initial mean absorbance value at 594 nm was approximately 0.533 and 1.511 for 2-day and 3-day biofilms, respectively [10], which were normalized to 1.0. In 2-day biofilms, treatment with 0.063 µg/ml of CLR (i.e. 4×MIC) caused a significant increase in biofilm biomass of approximately 1.2 fold (Fig. 2a). Moreover, the exposure of 3-day biofilms to 0.25 µg/ml CLR (i.e. 16×MIC) significantly increased the biofilm biomass, again by approximately 1.2 fold compared to the initial biofilm (Fig. 2b).

### Cell Viability of Biofilm Cells after Treatment of CLR

**Figure 3:**
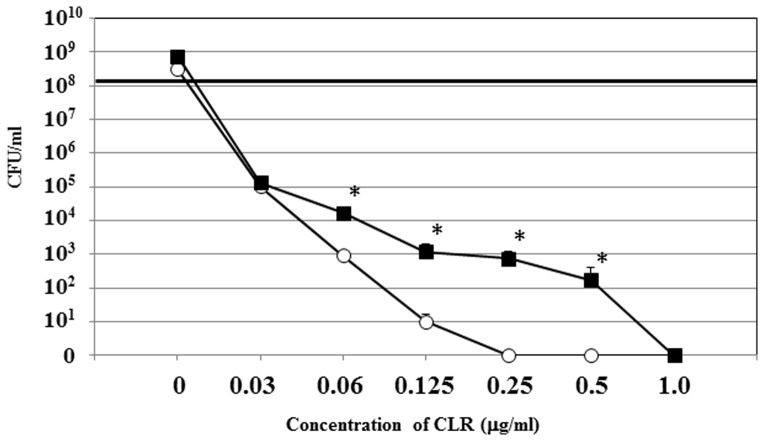
The effect of CLR on cell viability of the strain TK1402 biofilm. After exposure of 2-day biofilm (closed squares) and planktonic cells (open circles) with each concentration of CLR, viable cells were measured using CFU counting. The initial CFU for 2-day biofilm and planktonic cells adjusted to an optical density at 600 nm of 0.14 were approximately 0.3×10^8^ CFU. The CFU of CLR exposed biofilm or planktonic cells were measured. All of the results are expressed as the means ±1 standard deviation from at least three independent experiments. *significantly different (*p*<0.05) relative to CFU value (biofilm versus planktonic after treatment with the indicated concentrations of CLR concentration).

**Figure 4:**
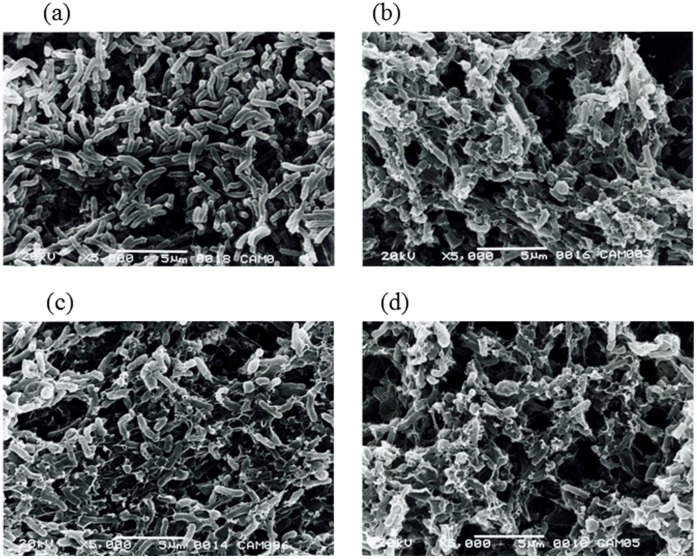
SEM images of TK1402 biofilm after various concentrations of CLR treatment. (a): the control cells (without treatment of CLR). (b): treated with 0.03 µg/ml of CLR. (c): treated with 0.06 µg/ml of CLR. (d): treated with 0.5 µg/ml of CLR. After treatment with the indicated concentrations of CLR, the biofilms were investigated using SEM. Scale bars are shown at the bottom of each electron microscope image.

The results indicating concentration-inversely dependent increase of the biofilm biomass after treatment with CLR might imply that the biofilm cells not only could survive but also grow in the presence of a relatively high concentration of CLR. However, crystal violet staining measures the total biofilm biomass but not cell viability [28]. To clarify whether the increasing biofilm biomass was due to cell growth, cell viability was measured by CFU counting of 2-day biofilms treated with serial dilution of CLR. Similarly treated 2-day planktonic cultures were also analyzed as a control. The CFUs of both the CLR exposed planktonic cultures and biofilms exhibited reduced cell viability (Fig. 3), compared to a slight increase in the untreated control plantonic and biofilm cells. When the cells were exposed to 0.25 µg/ml CLR, no viable colonies were detected in the planktonic cells whereas the biofilm cells could survive with an approximate 10^3^ CFU value. In addition, there was significantly increased viable cells in biofilms compared to planktonic cells after treatment with 0.06 µg/ml to 0.5 µg/ml CLR. However, there were no viable colonies when the biofilms were exposed to 1.0 µg/ml CLR. We also examined the CFU value of 3-day biofilms after exposure to CLR and the results were comparable to that of 2-day biofilms (data not shown). Further, we examined the cell morphology after 24 h of CLR treatment using a scanning electron microscope with 3-day biofilm (Fig. 4), since *H. pylori* can transform into a coccoid form, which is non-culturable but viable [29]. The control cells (without treatment of CLR) were composed primarily of cells with bacillary morphology which were clearly outlined (Fig. 4a). On the other hand, when the cells were treated with 0.03 µg/ml (Fig. 4b), 0.06 µg/ml (Fig. 4c) or 0.5 µg/ml (Fig. 4d) of CLR, almost all cells exhibited a rough outline and displayed damaged cell envelopes, although a few coccoid-like forms of cells were detected in the cells of CLR-treated biofilms. These results suggested that most of the cell mass increase in the biofilm represented dead cells. Nevertheless, Fig. 3 indicated that the minimum bactericidal concentration (MBC) of the biofilm cells to CLR was higher than that of planktonic cells (1.0 µg/ml vs. 0.25 µg/ml).

### Gene Expression of RND Efflux Pumps in Biofilms

**Figure 5:**
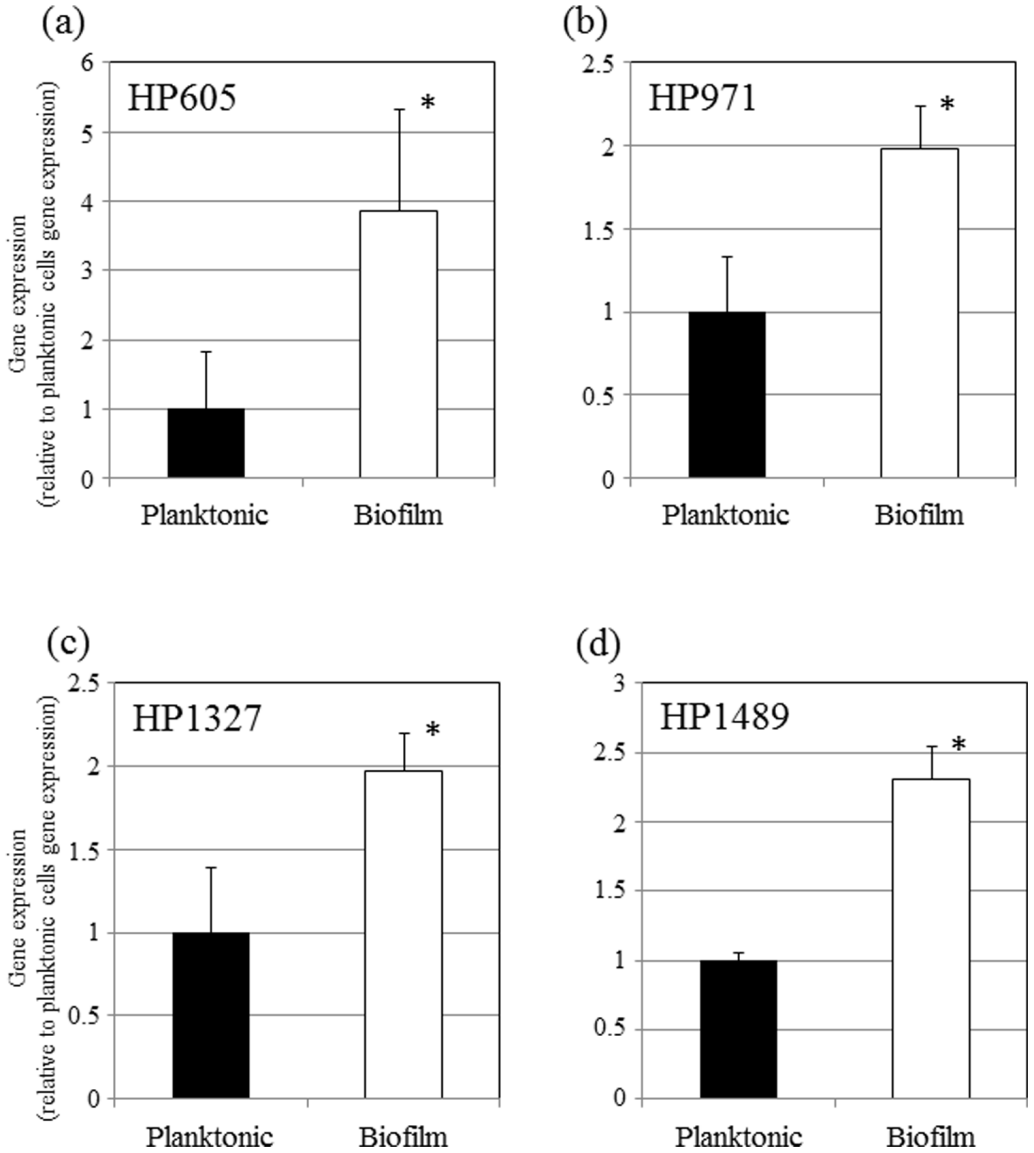
Expression of *H. pylori* efflux pump genes, HP605 (a), HP971 (b), HP1327 (c) and HP1489 (d). The quantity of cDNA corresponding to these genes was determined by real-time PCR and was normalized to that of the 16S rRNA gene in each unique reaction. Each experiment was repeated three times with at least duplicate samples from each independently isolated RNA preparation. Data are expressed as the means of all of experiments ± standard deviations. *significantly different (*p*<0.05) relative to the mRNA expression level (planktonic versus biofilm).

In *H. pylori*, the efflux pumps of the resistance-nodulation-cell division (RND) family are well described relative to their contribution to antibiotic resistance [26], [30], [31]. These reports indicated that four RND families have been identified in *H. pylori* (HP0605-HP607, HP0971-HP0969, HP1327-HP1329, and HP1489-HP1487). Therefore, we determined whether there were differences in the levels of transcription of these genes between biofilm and planktonic cells using specific primer pairs for HP605, HP971, HP1327, or HP1489 (primers sequences are described in Materials and Methods) with quantitative real-time RT-PCR (Fig. 5). It was revealed that the expression of these genes was significantly more elevated in the biofilm cells than in the planktonic cells. These results suggested that the high levels of these gene transcripts could contribute to biofilm resistance to CLR.

### Generation of Spontaneous CLR Resistant Mutations in Biofilms

**Figure 6:**
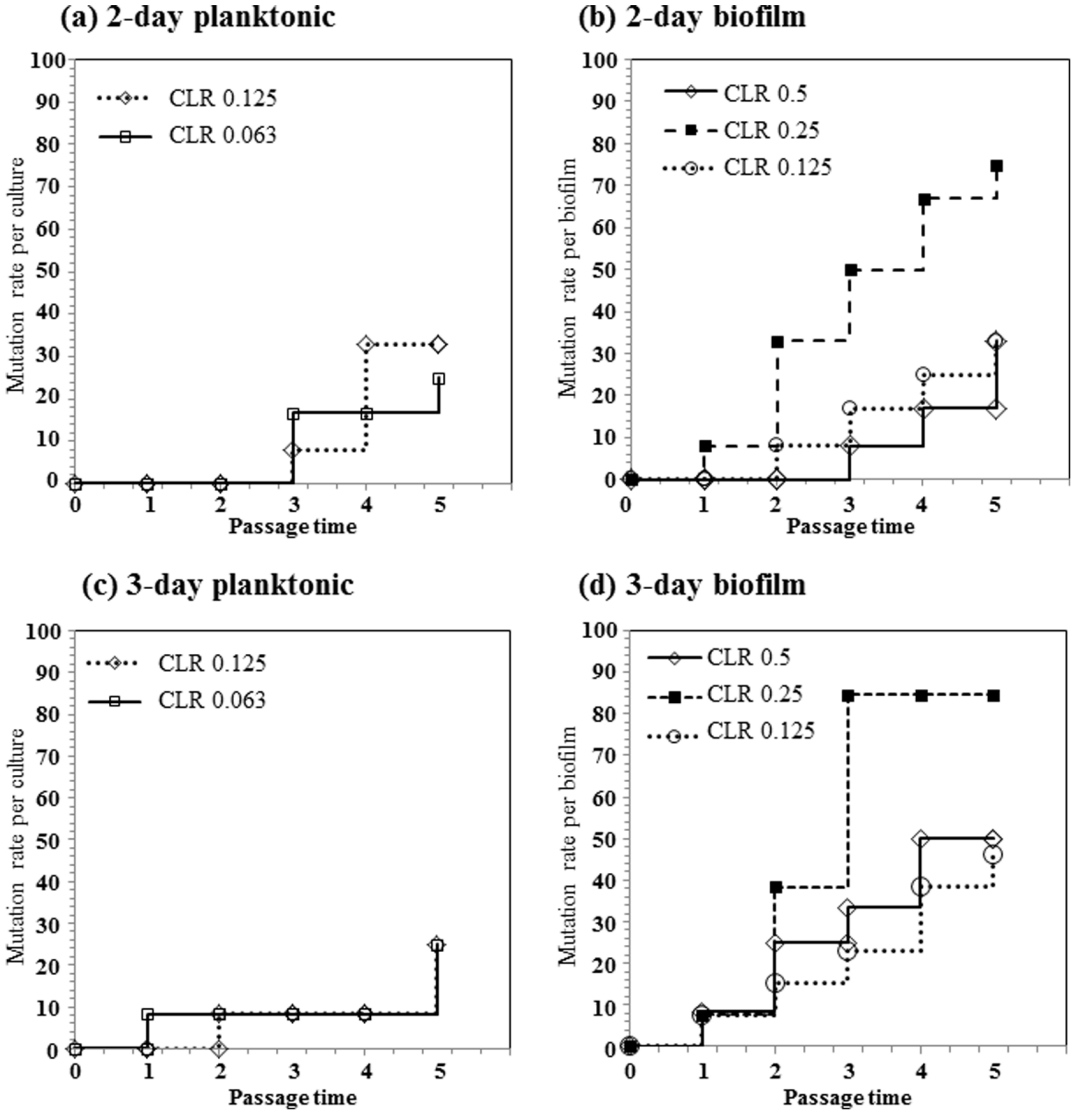
Induction of CLR resistance mutations in biofilm and planktonic cells. 2-day and 3-day biofilms (A and B) and planktonic cells (C and D) were exposed to each concentration of CLR. After an additional incubation for 24 h, cells were recovered in fresh Brucella-FCS agar, and the generation of CLR resistant mutants was assessed Brucella-FCS agar supplemented with 1.0 µg/ml CLR. When no CLR resistant cells were detected, exposure to CLR was repeated up to 5-times. The graph shows the accumulation ratio of the generated CLR resistant biofilm or planktonic cultures.

To examine the effect of biofilm formation by *H. pylori* on the generation of spontaneous resistant cells after exposure to CLR, the 2-day or 3-day biofilms were exposed to one-eighth, one-quarter or one-half of the MBC (Fig. 3) of CLR at concentrations of 0.125, 0.25, and 0.5 µg/ml, concentrations which are equivalent to 8×, 16×, and 32×MIC, up to 5 times or until a generation of CLR resistant cells was evident. As controls, 2-day or 3-day planktonic cultures were also exposed to one-quarter or one-half of the MBC (for planktonic cells, as shown in Fig. 3) of CLR at concentrations of 0.063 and 0.125 µg/ml, concentrations which are equivalent to 4× and 8× MIC. Fig. 6 shows the results of the accumulation ratio of the generated CLR resistant biofilm or planktonic cultures. In 2-day planktonic cultures, a few CLR resistant mutants were observed (25% (3/12) and 33% (4/12) at 0.063 µg/ml and 0.125 µg/ml of CLR, respectively) (Fig. 6a). In 3-day planktonic cultures, the generation of resistant cells was at a similar level as in 2-day planktonic cultures (Fig. 6c). In contrast, CLR resistant cells in biofilms were detected more frequently at 0.25 µg/ml (one-quarter MBC) CLR than that in control. Nine of twelve 2-day biofilm cells (75%) were CLR resistant (Fig. 6b), which increased to 84.6% (eleven of thirteen) in 3-day biofilms (Fig. 6d). Additionally, 3-day biofilms showed increased resistance at 0.5 µg/ml or 0.125 µg/ml CLR compared to controls (Fig. 6d), though there was no difference at these concentrations in 2-day biofilms.

### Sequence Analysis of 23S rRNA Mutations

All CLR resistant samples (54 samples in total) obtained in this study were examined for 23S rRNA point mutations. The primer pairs used (Hp23S 1942F and Hp23S 2308R), could detect the common mutations (at positions 2142 and 2143 of the 23S rRNA gene) associated with CLR resistance and sequence analysis of the PCR products was then carried out. All of the mutant strains showed a point mutation at either position 2142 (43 strains) or 2143 (11 strains) of the 23S rRNA gene. At position 2142, 41 strains showed an A to G transition. The remaining 2 strains were: a strain from a 2-day biofilms after the third exposure to 0.5 µg/ml CLR exhibiting an A to T transition and a 2 day planktonic culture after the third exposure to 0.125 µg/ml CLR exhibiting an A to C transition. In the mutations at position 2143, all strains showed an A to G transition.

## Discussion

In this study, we determined that biofilm formation in *H. pylori* increased the resistance to CLR at MIC levels by up to 4-fold in 2-day biofilms and to 16-fold in 3-day biofilms as well as MBC levels by up to 4-fold compared to planktonic cells (Fig. 1, Fig. 2, and Fig. 3). Similar phenomena of increased resistance to antibacterial agents have been reported in other biofilm forming bacteria [2], [32], [33]. In other bacterial species, multiple mechanisms of biofilm resistance to antimicrobial compounds were suggested (i) failure of the antimicrobial compounds to penetrate the biofilm, (ii) slow growth of the biofilm cells owing to nutrient limitation, (iii) activation of the general stress response [32], [33], [34], [35], [36], [37]. Our present data showed that an increase in the biofilm biomass was observed after treatment with CLR (Fig. 2). However, the viability of the CLR treated biofilm cells was reduced in a dose dependent manner (Fig. 3). We hypothesize that these observations might reflect the time needed for CLR to diffuse because of the presence of the biofilm extracellular matrix (equivalent to (i) mentioned above). We previously demonstrated that the OMV produced by *H. pylori* strain TK1402 plays an important role in the formation of the extracellular matrix of the biofilm [10]. In addition, several studies indicated that the presence of extracellular DNA and mannose–related proteoglycans can contribute to the formation of biofilms as extracellular matrix components [38], [39]. The extracellular matrix may exhibit a sequestering effect on CLR relative to internal cells within the biofilm. As a result, the biofilm biomass is increased after treatment with CLR but with time CLR diffuses to the interior of the biofilm followed by a decrease in cell viability. However, little is currently known regarding biofilm resistance in this microorganism and other mechanisms may also contribute to resistance. Specifically, participation of the efflux pumps of the RND family concerned with the development of antibiotic resistance has been well studied in *H. pylori* [26], [30], [31]. We analyzed the expression of mRNA for the efflux pumps genes (HP605, HP971, HP1327, or HP1489), and the expression of these genes was significantly more elevated in the biofilm cells than in the planktonic cells (Fig. 5). These results suggested that the high level of these genes transcript could contribute to biofilm resistance to CLR. To further test the potential contributions of other mechanisms, we analyzed the susceptibility of planktonic cultures at early exponential phase and stationary phase to CLR using a culture method and the late stationary phase cells were more resistant at 0.06 µg/ml than early exponential phase cells (data not shown). This result suggested indirectly that the slow growth of *H. pylori* cells might reduce the antimicrobial activity of CLR. Taken together, these observations suggested that there are multiple resistance mechanisms that could account for *H. pylori* biofilm cell resistance to CLR. Further characterization will be required to delineate the resistance mechanisms of biofilm cells.

In the previous *H. pylori* whole genome analysis, two copies of the 23S rRNA gene were detected in this microorganism [40], [41], [42]. We determined the properties of the two copies of the 23S rRNA gene in the strain TK1402 chromosome using Southern blotting (data not shown). When we examined the mutation sites of 23S rRNA by sequencing analysis, only one nucleotide was identified at positions 2142 or 2143 in the 23S rRNA of all samples. If only one copy of the 23S rRNA gene was mutated, equal amounts of PCR products from the mutated and wild-type copies should be amplified, indicating that both copies of the 23S rRNA at position 2142 or 2143 were mutated in all CLR resistant strains generated in this study. Taylor et al. reported that the majority of CLR resistant *H. pylori* require mutations in both copies of the 23S rRNA gene to confer CLR resistance [40], and this is consistent with our sequencing results.

Previous reports have indicated that mutations related to CLR resistance are generated at a very low frequency during *in vitro* CLR passage [43], [44]. On the other hand, it is obvious that CLR resistance mutations were frequently generated in our present study, especially during exposure to 0.25 µg/ml of CLR, where the rate was 75% and 85% in 2-day and 3-day biofilms, respectively (Fig. 6b and 6d). The highly effective generation of CLR resistant mutants may be related to the concentration of CLR used in addition to the formation of biofilms. CLR concentrations used in previous reports were around one-half of the MIC but we were able to use one-eighth to one-half the MBC concentration of CLR (equivalent to 8- to 32-fold the MIC of strain TK1402). This relatively high concentration, especially one-quarter of MBC (0.25 µg/ml) of CLR may facilitate the generation of CLR resistance mutations. Further research is now in progress to examine this possibility.

CLR is well distributed throughout the human body and achieves high concentrations in tissue. Nakamura et al. reported that CLR concentrations in gastric juices, mucosa or serum after administration of 500 mg of the drug for 7 days were 550.6, 64.6 or 2.5 µg/ml at 2 hours after administration, and 43.4, 36.2 or 2.2 µg/ml at 6 hours, respectively [45]. These concentrations were sufficient to reduce the levels of *H. pylori* in vivo so that this microorganism formed biofilms. However, to reach such high concentrations of CLR in gastric mucosa for extended periods, the drug needs to be taken with sufficient dosage. In addition, in cases with inadequate compliance with eradication therapy, the concentration of CLR does not reach high concentrations in the gastric mucosa. Further, macrolides including CLR are frequently used in the treatment of various infectious diseases in pediatric, respiratory and otorhinolaryngology settings. In these cases, biofilm formation by *H. pylori* may contribute to the acquisition of CLR resistance. There are few studies in the literature regarding the relevance of mutational events in biofilm antibiotic resistance [46], [47]. To our knowledge, this is the first demonstration that biofilm formation can affect the generation of antibiotic resistance mutations in *H. pylori*.

In some countries including Japan, triple therapy containing CLR is the best option for eradication of *H. pylori*. CLR resistance in *H. pylori* has serious implications for first-line eradication therapy in such countries, since it is thought to be the major factor in eradication failure, although other antibiotics such as amoxicillin were prescribed [15]. Our present study demonstrated that the biofilm forming ability of *H. pylori* contributes to the development of CLR resistance and increases the frequency of the development of CLR resistant mutants relative to planktonic cells. However, since susceptibility to antibiotics has traditionally been evaluated using planktonic cells, and previous studies have shown that *H. pylori* forms biofilm on human gastric mucosa [7], [8], [9], these MICs are not reliable predictors of the antibiotic effects in the human stomach. CLR resistance in *H. pylori* can therefore be acquired by the selection of spontaneous mutation events that occur due to the magnitude and duration of macrolide use on the human gastric mucosa. Recently, a clinical trial for effective strategies targeting *H. pylori* biofilm infection through the use of molecules such as *N*-acetylcysteine was reported, and the eradication rate was increased compared to that of a non-treated group [48]. This study and our present results suggest that the assessment of the ability to form biofilms in *H. pylori* could play an important role in preventing and controlling the generation of antibiotic resistance.
